# Enhancing Efficiency in Trustless Cryptography: An Optimized SM9-Based Distributed Key Generation Scheme

**DOI:** 10.3390/s24247874

**Published:** 2024-12-10

**Authors:** Jinhong Chen, Xueguang Zhou, Wei Fu, Yihuan Mao

**Affiliations:** 1 Information Security Department, Naval University of Engineering, Wuhan 430033, China; 21101003@nue.edu.cn (J.C.); 0909051003@nue.edu.cn (X.Z.); myhhz1@163.com (Y.M.); 2 College of Economics and Trade, Jiangxi Vocational College of Finance and Economics, Jiujiang 332000, China

**Keywords:** intelligent system, distributed key generation, identity-based cryptography, share transformation protocol, SM9

## Abstract

Intelligent systems are those in which behavior is determined by environmental inputs, and actions are taken to maximize the probability of achieving specific goals. Intelligent systems are widely applied across various fields, particularly in distributed intelligent systems. At the same time, due to the extensive interaction with user data, intelligent systems face significant challenges regarding security. This study proposes an optimized distributed key generation (DKG) scheme for identity-based cryptography (IBC) using the SM9 standard. Our scheme introduces a (t, n)-threshold system that functions without a trusted center, addressing the vulnerability of single points of failure in conventional key generation centers (KGCs). We reduce communication and computational demands by refining the Paillier share transformation protocol, ensuring efficient, centerless operations. The scheme’s security, validated in the existential unforgeability against adaptive chosen identity attacks (EUF-CIA) model, demonstrates its practical applicability and enhanced security for distributed intelligent systems.

## 1. Introduction

Intelligent systems are those in which behavior is determined by environmental inputs, and actions are taken to maximize the probability of achieving specific goals. Intelligent systems are widely applied across various fields [1], such as transportation [2], healthcare [3,4], smart homes [5], and the Internet of Things, providing more efficient and intelligent services to humanity [4]. However, the integration of different technologies and extensive interaction with user data also creates opportunities for malicious activities, posing significant challenges to the security of intelligent systems [1]. In recent years, significant research has focused on the role of intelligent systems in enhancing cybersecurity. There is an increasing discourse around the applications of artificial intelligence (AI), machine learning, and deep learning within the field of cybersecurity [6,7]. Authentication technology is particularly critical in this context, especially within distributed intelligent systems, where ensuring identity security becomes a top priority.

The distributed key generation (DKG) technique, a multifaceted cryptographic protocol, engages numerous participants with the primary objective of bolstering the security and dependability of network applications. This property is achieved through the collaborative and secure generation and management of cryptographic keys in a distributed architecture. The strategic allocation and storage of key fragments across multiple key generation centers (KGCs), facilitated through collective negotiation, markedly reduces the vulnerabilities associated with single-point failures and the potential for privileged misuse. In contemporary practice, DKG methods have been extensively applied to facilitate secure communications among various users, underpinning their significance in the current digital communication context.

Pedersen [8] introduced a pioneering DKG protocol designed for discrete logarithm-based threshold cryptographic systems, which has also been used as a subprotocol in cryptographic signature and decryption computations. Notably, the initial protocol lacked a comprehensive elucidation of the security framework of the protocol. This gap was addressed by Gennaro et al. [9], who not only identified and articulated the security shortcomings of Pedersen’s approach but also developed and rigorously proved the security of an enhanced DKG protocol. This novel protocol advances security measures without compromising operational efficiency. Furthermore, Gennaro et al. [10] extended their study by integrating Pedersen’s protocol within Schnorr [11] signatures.

In a major stride towards practical deployment, Kate and Goldberg [12] unveiled the inaugural application of the DKG framework in an internet setting by establishing a proven secret sharing mechanism. Concurrently, Fouque and Stern [13], Zhang and Imai [14], and Canny and Sorkin [15] explored noninteractive and scalable DKG protocols. Zhang and Wang [16] introduced a versatile DKG protocol based on a discrete logarithmic public key infrastructure. Characterized by minimal data requirements for secret preservation, assured randomness, and uniform private key distribution, this protocol can be distinguished because of its various applications in group cryptography beyond conventional threshold structures.

To further advance the field, Zhang [17] proposed a DKG protocol grounded in information-theoretic security principles that featured a verifiable secret sharing scheme adapted to vector space access structures and complete security proof. Zha et al. [18] developed an adaptive DKG scheme that facilitates dynamic membership changes, thereby enhancing system efficiency. Zhang and Zhang [19] focused on bilinear group applications, and Wang et al. [20] introduced a DKG algorithm designed for the Lewko–Waters identity encryption protocol, thereby significantly bolstering the resilience and robustness of its key management system. Lindell and Nof [21] proposed an elliptic curve digital signature algorithm (ECDSA)-based DKG protocol by incorporating zero-knowledge proofs to safeguard against dishonest participation. Finally, Gennaro and Goldfeder [22] introduced a threshold DKG protocol within ECDSA by employing a homomorphic share transformation protocol to potentially minimize participant numbers.

In 1984, Shamir [23] introduced a groundbreaking identity-based cryptosystem, demonstrating a seminal advancement in identity-based cryptography (IBC). This system innovatively employs a unique identifier as the public key, thereby greatly streamlining the key management process by eliminating the conventional key distribution paradigm inherent in traditional public key cryptography. In this framework, a user’s public key is algorithmically derived from a unique identifier, thereby obviating the need for a certifying authority to validate the authenticity of the public key. This mechanism enhances the security profile of the system by centralizing the generation and distribution of private keys to the users through the KGC [24], although the notion of private keys being exclusive to the user is thus redefined. Despite the efficacy of IBC in bolstering security within zero-trust network environments, the secure deployment of such a public key infrastructure poses considerable challenges [25].

In 2016, the advent of the identity-based cryptographic algorithm SM9, released by the China Cryptography Administration [26], marked a significant milestone in cryptographic standards. Notably, the digital signature algorithm component of SM9 has been recognized and adopted by the International Organization for Standardization (ISO)/International Electrotechnical Commission (IEC) international standards, underscoring its global acceptance and reliability. As the sole identity cipher algorithm standard emanating from China, SM9 plays a pivotal role in safeguarding the integrity and confidentiality of information systems across various sectors by handling commercial secrets within the country [27]. The widespread deployment of SM9 identity-based cryptographic algorithms across numerous domains attests to their foundational contribution to ensuring the security of China’s autonomously controlled infrastructure networks.

Recent studies have explored various facets of the SM9 DKG scheme, yielding noteworthy advancements and identifying persistent challenges (Table 1). Ma [28] introduced a threshold DKG protocol by leveraging the SM9 algorithm, which is notable for its requirement of a relatively high number of participants. Similarly, Zhang et al. [25] developed an SM9-based threshold protocol and encountered analogous concerns regarding participant numbers. Tu et al. [29] investigated the domain of homomorphic secure multi-party computation to propose an SM9 threshold protocol that can accommodate various application scenarios within the threshold DKG framework. However, reliance on homomorphic encryption introduces a significant computational overhead, thereby limiting its practicality. Conversely, Yu et al. [30] presented a distributed identity cryptography management scheme that, although innovative, is constrained to bipartite protocol implementation, thus restricting its broader applicability.

In the context of SM9 DKG, where t denotes the number of KGCs actively participating in the key generation process and n denotes the total number of KGCs with secret shares, our analysis reveals a critical observation. Among the various schemes reviewed, only the one proposed by Tu et al. [30] achieves the optimal threshold value (t), which is a crucial metric for system security and reliability. However, this achievement comes at a significant cost, thus rendering the system impractical for widespread deployment due to excessive overheads. In contrast, our proposed scheme attains this optimal threshold value and introduces substantial reductions in the system costs. These two advantages significantly enhance the practicability of our scheme, positioning it as a more viable solution for real-world applications.

The main contribution of this study concerns an enhanced (t,n)-threshold DKG scheme based on SM9, devoid of reliance on trusted centers, called the SM9-based lightweight DKG scheme. Our innovative approach significantly mitigates the communication overhead and computational demands of the Paillier homomorphic encryption and decryption processes by refining the Paillier share transformation protocol while maintaining the optimal threshold value. Furthermore, we rigorously demonstrate the security of our scheme within the existential unforgeability against adaptive chosen identity attack (EUF-CIA) model, providing a formal security proof that establishes the reduction in our scheme to the τ-BCAA1 problem under the random oracle model.

## 2. Preliminaries

### 2.1. Shamir Threshold Secret Sharing Scheme

**Figure 1:**
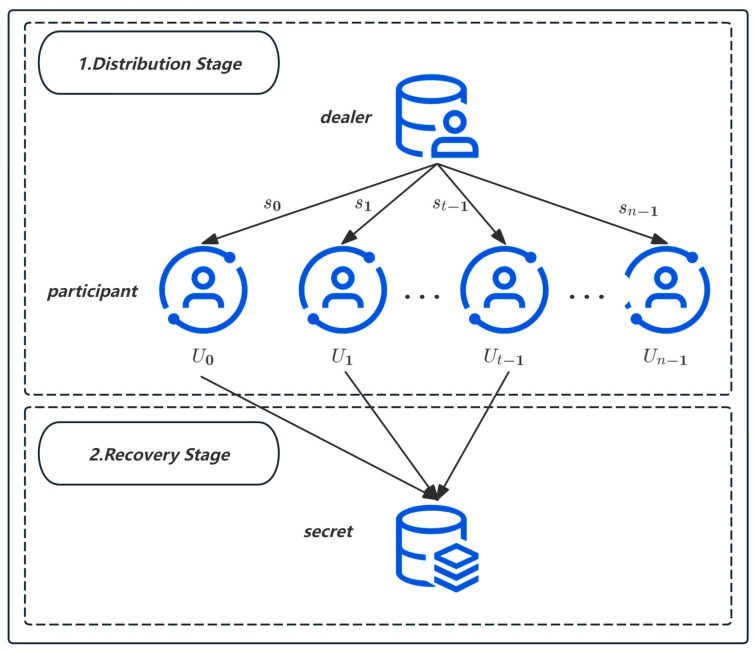
Shamir Threshold Secret Sharing Flowcharts.

The Shamir threshold secret sharing scheme, introduced by Shamir et al. in 1979, is a foundational cryptographic protocol designed to distribute and reconstruct secrets securely. It operates on the principles of the Lagrange interpolation theorem and can be delineated into two primary phases. Figure 1 shows the flowchart of the Shamir threshold secret sharing scheme.

1. Distribution Stage

In this initial phase, the dealer, aiming to securely share a secret, first selects arbitrary coefficients $a_{i}$ from the multiplicative group $Z_{N}^{*}$ for i=0 to t−1, where t denotes the threshold number of participants required for secret reconstruction. The dealer then chooses n distinct, non-zero elements $x_{i}$ from $Z_{N}^{*}$, formulating a polynomial $f\left(x\right)=\sum_{i=0}^{t-1}a_{i}x^{i}$, where $a_{0}$ represents the secret. Subsequently, the dealer computes the shares $s_{i}=f\left(x_{i}\right)$ for each participant $U_{i}$, distributing them accordingly.

2. Recovery Stage

During the reconstruction phase, any group of t participants can collaboratively recreate the original polynomial $f\left(x\right)$ utilizing the Lagrange interpolation theorem. By setting x=0, the secret $a_{0}$ is revealed through the formula: $a_{0}=\sum_{i=0}^{t-1}s_{i}\prod_{j=0\cap j\neq i}^{t-1}\frac{x_{j}}{x_{j}-x_{i}}$.

### 2.2. Paillier Homomorphic Encryption Algorithm

The Paillier homomorphic encryption algorithm, predicated on the complexity of the residual class problem, is a pivotal cryptographic scheme for encrypted data processing. This algorithm encompasses three principal steps:

1. Key Generation
  (a) Begin by selecting two large prime numbers p and q of identical length, ensuring that $gcd\left(pq,\left(p-1\right)\left(q-1\right)\right)=$**1**, where gcd represents the Greatest Common Divisor.
  (b) Calculate n=pq and $\lambda =lcm\left(p-1,q-1\right)$, where lcm represents the Least Common Multiple.
  (c) Set g=n+1, and define the function $L\left(x\right)=\frac{x-1}{n}$. Compute $\mu ={\left(L\left(g^{\lambda }mod\;n^{2}\right)\right)}^{-1}mod\;n$, where mod represents the modulus operation.
  (d) The public key is given by $\left(n,g\right)$, and the private key by $\left(\lambda ,\mu \right)$.
2. Encryption
  (a) For a message m within the range $\left[0,n\right]$, select a random number r from $Z_{n}^{*}$ where $gcd\left(r,n\right)=1$.
  (b) Compute the ciphertext c as $c=g^{m}r^{n}mod\;n^{2}$.
3. Decryption
  (a) Given ciphertext c, confirm $c\in Z_{n^{2}}^{*}$.
  (b) Derive the message m as $m=L\left(c^{\lambda }mod\;n^{2}\right)\cdot \mu \;mod\;n$.

The Paillier algorithm is distinguished by its capacity for additive and scalar multiplication homomorphic encryption, delineated as follows:

1. Addition homomorphism

For ciphertexts $c_{1}$ and $c_{2}$, the encrypted sum is $c_{1}*c_{2}={Enc}_{pk}\left(m_{1}+m_{2}\right)$, and hence, ${Dec}_{pk}\left(c_{1}*c_{2}\right)={Dec}_{pk}\left({Enc}_{pk}\left(m_{1}+m_{2}\right)\right)=m_{1}+m_{2}$

2. Scalar multiplication homomorphism

For a ciphertext $c_{1}$ and a scalar k, the operation ${c_{1}}^{k}={Enc}_{pk}{\left(m_{1}\right)}^{k}$ allows for the encrypted product to be decrypted as ${Dec}_{pk}\left({c_{1}}^{k}\right)={Dec}_{pk}\left({Enc}_{pk}{\left(m_{1}\right)}^{k}\right)=k\;*\;m_{1}$

### 2.3. Paillier-Based Share Conversion Protocol

This protocol facilitates two parties, Alice and Bob, who possess multiplicative shares $a,b\in Z_{n}$ of a secret, such that x=ab mod n, in securely converting these shares into additive shares α+β=x. The steps are meticulously designed to maintain confidentiality throughout the process.

1. Initial encryption and share transmission

Alice begins by encrypting her share a using her public key, resulting in $c_{a}={Enc}_{pk}\left(a\right)$. She then transmits $c_{a}$ to Bob.

2. Bob’s calculation and transmission

Upon receiving $c_{a}$, Bob computes $c_{b}={c_{a}}^{b}\;*\;{Enc}_{pk}\left(\beta^{\prime }\right)={Enc}_{pk}\left(ab+\beta^{\prime }\right)$, where $\beta^{\prime }\in_{R}Z_{n}$. Bob deduces his additive share of the secret as $\beta =-\beta^{\prime }$ and forwards $c_{b}$ to Alice.

3. Alice’s decryption and share derivation

Alice, upon receiving $c_{b}$, decrypts it to ascertain her additive share of the secret, $\alpha ={Dec}_{sk}\left(c_{b}\right)$.

This protocol effectively enables Alice and Bob to transition from holding multiplicative shares of a secret to possessing additive shares, thereby facilitating various cryptographic operations while preserving the privacy of their inputs.

### 2.4. SM9 Digital Signature Algorithm: An Overview

The SM9 digital signature algorithm, anchored in the principles of bilinear pairings, delineates a comprehensive procedure for secure digital signing and verification. It unfolds through the following stages:

1. System setup
  (a) Initialization is performed by inputting safety parameters k, two additive cyclic groups $G_{1},$ $G_{2}$ of prime order N, and one multiplicative cyclic group $G_{T}$ also of prime order N, ensuring these groups satisfy a bilinear mapping $e:G_{1}\times G_{2}\to G_{T}$. The groups $G_{1},$ $G_{2}$ are defined by their generators $P_{1},P_{2}$, respectively.
  (b) Define two hash functions $H_{1}:{\left\{0,1\right\}}^{*}\to Z_{N}^{*},H_{2}:{\left\{0,1\right\}}^{*}\to Z_{N}^{*}$.
  (c) The KGC selects a master private key $msk\in Z_{N}^{*}$, computes the master public key $mpk=msk\;*\;P_{2}$, and selects a signature private key generation function identifier hid.
  (d) KGC broadcasts the public system $param=<G_{1},G_{2},G_{T},P_{1},P_{2},mpk,N,H_{1},H_{2},hid>$ and securely stores msk.
2. User signature private key extraction
  (a) KGC computes $t_{1}=(H_{1}\left({ID}_{A}|\left|hid,N\right)+msk\right)mod\;N$. If $t_{1}=0$, KGC retries with a different msk. Otherwise, it proceeds by calculating $t_{2}=msk\cdot t_{1}^{-1}modN$.
  (b) The signature private key $ds=t_{2}\;*\;P_{1}$ is then computed and sent to the user identified by ${ID}_{A}$.
3. Digital signature creation
  (a) The signer computes $g=e\left(P_{1},mpk\right)$.
  (b) A random positive integer $r\in_{R}Z_{N}^{*}$ is selected to compute $w=g^{r}$.
  (c) The hash $h=H_{2}(Msg||w,N)$.
  (d) The signer computes $l=\left(r-h\right)modN$. If l=0, r is reselected; otherwise, the signature S=l ∗ ds is formed, with $\left(h,S\right)$ constituting the digital signature of Msg.
4. Verification
  (a) The verifier, equipped with system parameters, user identification ${ID}_{A}$, message ${Msg}^{\prime }$, and digital signature $\left(h^{\prime },S^{\prime }\right)$, first checks if $h^{\prime }\in Z_{N}^{*}$ and $S^{\prime }$ is a point in $G_{1}$.
  (b) Calculations are performed to validate the signature: compute $t=g^{h^{\prime }}$, $h_{{ID}_{A}}=H_{1}=({ID}_{A}||hid,N)$, $P=h_{{ID}_{A}}P_{2}+mpk$, and $u=e\left(S^{\prime },P\right)$, then w=u ∗ t.
  (c) If $h^{\prime }=H_{2}({Msg}^{\prime }||w,N)$, the signature is verified successfully; otherwise, the verification fails.

## 3. SM9-Based Lightweight Distributed Key Generation Scheme

In this section, we focus on the formal definition of a scheme, the description of a scheme, and scheme security.

### 3.1. System Model

**Figure 2:**
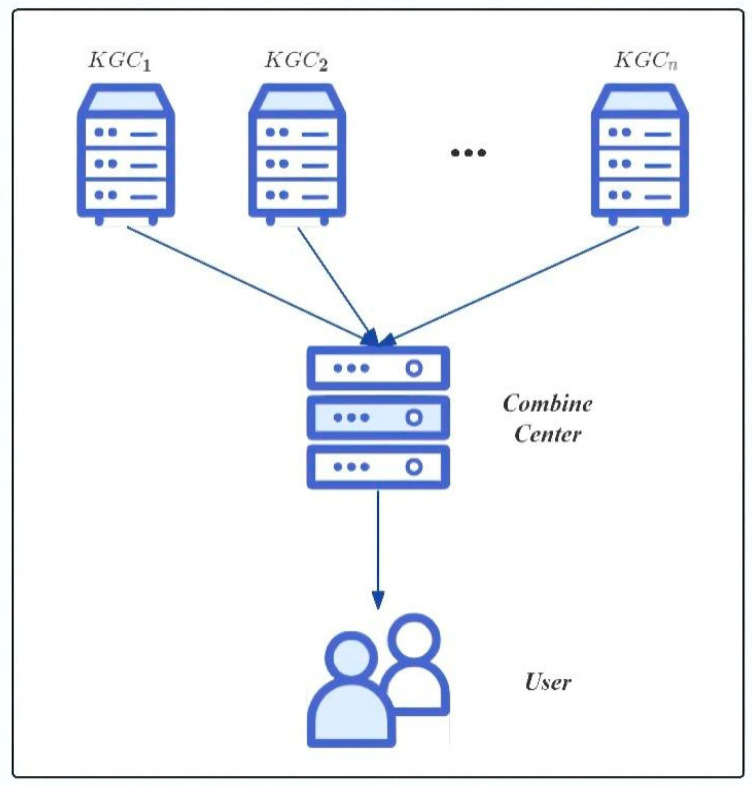
Distributed key generation system model.

In the proposed scheme, depicted in Figure 2, we delineate a system comprising the following three pivotal entities: the KGC, Combine Center, and User. These entities are foundational to the architecture, each playing a unique role.

1. Key generation center (KGC)

Entrusted with generating segments of both the master public and private keys, the KGC is integral to facilitating secure and distributed cryptographic operations. It operates within a collaborative network of centers in which each can securely produce valid signature private key fragments for users based on their unique identifiers (IDs). This distributed mechanism significantly bolsters the security of the system, mitigating risks associated with centralized key management frameworks and underscoring the central role of the KGC in preserving the integrity and confidentiality of the cryptographic process.

2. Combine center

Tasked with the critical role of amalgamating signature private key fragments into a singular, valid signature private key, the operations of the Combine Center are marked by precision and stringent security measures to thwart unauthorized access or tampering. Upon successful assembly of the signature private key, it is securely dispatched to the designated user, thereby facilitating the execution of cryptographic operations without imposing the technical complexities of key generation upon the end user.

3. User

Positioned as the endpoint in our cryptographic narrative, the user, encompassing a broad spectrum of devices within the Internet of Things (IoT) ecosystem, is the beneficiary and operator of the signature private key. This role extends beyond human interaction to include an array of devices by leveraging the secure infrastructure established by the KGC and Combine Center for authenticated and encrypted communications.

### 3.2. Scheme Definition

This subsection outlines the architecture of the SM9-based lightweight DKG scheme, incorporating advancements from prior studies. Key features include a distributed configuration, private key derivation, and amalgamation processes, detailed as follows.

1. Distributed setup (DSetup)

(1) $$\left({msk}_{i},{mpk}_{i},{sk}_{i},{pk}_{i}\right)\leftarrow DSetup\left(t,n,\varrho \right)$$

Given parameters t,n, and ϱ, where t represents the threshold number, n is the number of Key Generation Centers (KGCs), and ϱ denotes the public system parameter, the distributed setup generates:

- ${msk}_{i}$: The private key slice for ${KGC}_{i}$,
- ${mpk}_{i}$: The public key fragment for ${KGC}_{i}$,
- ${sk}_{i},{pk}_{i}$: The Paillier key pair for ${KGC}_{i}$.

2. Distributed private key extraction (DExtract)

(2) $$\left({ds}_{i})\leftarrow DExtract(ID,{msk}_{i}\right)$$

For a user with identifier ID, this process extracts ${ds}_{i}$, representing the user’s private key share from each ${KGC}_{i}$, utilizing their respective ${msk}_{i}$.

3. Combine private key

(3) $$\left(ds\right)\leftarrow Combine\left({ds}_{i}\right)$$

The user’s private key, ds, is synthesized from the individual shares ${ds}_{i}$, effectively combining them into a singular private key.

### 3.3. Security Definition and Security Models

Here, we introduce a security framework paralleling the EUF-CMA model for digital signatures, adapted to evaluate the robustness of our scheme under stochastic predicate analysis.

**Assumption 1:**  Bilinear Collision Attack Assumption (τ-BCAA1).

ψ is an isomorphism from $G_{2}$ to $G_{1}$ with $\psi \left(P_{2}\right)=P_{1}$. Positive integer τ and $\alpha \in_{R}Z_{N}^{*},{P_{1}\in G_{1},P}_{2}\in G_{2},e:G_{1}\times G_{2}\to G_{T}$, as we know $\left(P_{1},P_{2},\left[\alpha \right]P_{2},h_{0},\left(h_{1},\left[\frac{\alpha }{h_{1}+\alpha }\right]P_{1}\right),\ldots ,\left(h_{\tau },\left[\frac{\alpha }{h_{\tau }+\alpha }\right]P_{1}\right)\right)$, where $h_{1}\in_{R}Z_{N}^{*}$ and distinct for 0≤i≤τ, computing ${e\left(P_{1},P_{2}\right)}^{\frac{\alpha }{h_{0}+\alpha }}$ is hard.

**Definition 1:**  Existential Unforgeability Against Adaptive Chosen Identity Attacks (EUF-CIA): a distributed key generation scheme is deemed to exhibit existential unforgeability against adaptive chosen identity attacks (EUF-CIA) if, for any polynomial-time adversary, the advantage in succeeding in the defined security trials is negligible.

The security experiment is formalized through a game played by a challenger and an adversary. In the game, the adversary fixes $\left|A\right|\leq t-1$ semi-honest KGCs and the challenger simulates $\left|B\right|=n-t$ honest KGCs.

**Security Experiment:**  The robustness of the scheme against EUF-CIA is assessed through a structured game involving a challenger and an adversary, detailed as follows:

**Setup Phase:**  The challenger and the adversary collaboratively execute the distributed setup algorithm. By the conclusion of this phase, both parties acquire all master public keys (mpk) and their respective private master key slices (${msk}_{i}$).

**Phase 1 (Query Phase):**  The adversary is permitted to adaptively request DExtract for identities of its choosing. For each query on identity ${ID}_{i}$, the challenger engages in the DExtract and Combine Private Key processes with the adversary, subsequently providing the adversary with the private key share ${ds}_{i}$.

**Forgery Phase:**  The adversary attempts to forge private key shares ${ds}_{i}$ for an identity ID and is considered successful if:

- The combined private key ds, derived from ${ds}_{i}$ using the Combine operation, is valid for the identity ID.
- The private key for ID was not previously queried in Phase 1.

The advantage ϵ of winning the game is the probability of returning a valid forged private key.

### 3.4. Scheme Description

Our enhancement of the SM9 framework focuses on optimizing the digital signature and user-specific private key generation by employing the following innovative design principles.

Lagrange secret sharing is used to generate the master public key fragment msk by all the KGCs in the network so that the master public key $mpk=msk\;*\;P_{2}$ can be calculated.

The SM9 signature private key equation is expanded as follows:

(4) $$ds=\frac{msk}{msk+h}P_{1}=P_{1}-\frac{h}{msk+h}P_{1}=P_{1}-{\left(msk+h\right)}^{-1}hP_{1}$$

Using the Pallier share transformation protocol, the ${\left(msk+h\right)}^{-1}P_{1}$ can be calculated without revealing the respective secrets generated by the KGC; then, the user’s signature private key can be calculated.

The steps are as follows:

1. Distributed setup
  (a) ${KGC}_{i}$ uses the recommended values in the SM9 state secret standard for system parameter generation.
  (b) ${KGC}_{i}$ generates the respective Paillier public-private key pairs ${{(pk}_{i},sk}_{i})$, then broadcasts ${pk}_{i}$.
  (c) ${KGC}_{i}$ selects a random polynomial of degree ***t*** − 1, $f_{i}\left(x\right)=\sum_{j=0}^{t-1}a_{ij}\;*\;x^{j}$, where $a_{i0}$ is the secret share chosen by ${KGC}_{i}$. It broadcasts $C_{ij}=a_{ij}P_{2},i\in \left[1,n\right]\&j\in \left[1,t-1\right]$.
  (d) ${KGC}_{i}$ calculates $f_{i}\left(x_{j}\right)$, where $x_{j}$ is the secure hash value of ${KGC}_{j}$’s identity. It secretly sends the calculation result $f_{i}\left(x_{j}\right)$ to ${KGC}_{j}$.
  (e) After receiving $f_{j}\left(x_{I}\right)$ from ${KGC}_{j}$, ${KGC}_{i}$ verifies that $\sum_{j=0}^{t-1}C_{ij}\;*\;x_{i}^{j}=f_{j}\left(x_{i}\right)*P_{2}$. If the verification passes, it indicates that ${KGC}_{j}$ has not cheated and moves to the next step.
  (f) All ${KGC}_{i}$ can calculate the master public key $mpk=\sum_{i=1}^{n}C_{i0}=\sum_{i=1}^{n}a_{i0}P_{2}$.
2. Distributed private key extract
  (a) ${KGC}_{i}$ calculates the main private key shard, a t-threshold private key share $k_{i}=\sum_{i=1}^{n}f_{j}\left(x_{i}\right)$.
  (b) ${KGC}_{i}$ calculates the hash value of user identity $h=H_{1}({ID}_{A}||hid,N)$.
  (c) ${KGC}_{i}$ randomly chooses $\gamma_{j}\in_{R}Z_{N}^{*}$ and invokes the Paillier share conversion protocol to calculate:
  (d) $k^{-1}hP_{1}={\left(\sum_{i=0}^{t-1}k_{i}^{\prime }\sum_{j=0}^{t-1}\gamma_{j}\right)}^{-1}\sum_{j=0}^{t-1}\gamma_{j}hP_{1}={(\sum_{i=0}^{t-1}\delta_{i})}^{-1}\sum_{j=0}^{t-1}\gamma_{j}hP_{1}$, where the denominator part of the user signature private key $k=\sum_{i=0}^{t-1}k_{i}^{\prime }=\sum_{i=0}^{t-1}\left(\lambda_{i}k_{i}+\frac{h}{t}\right)=\sum_{i=0}^{t-1}a_{i0}+h=msk+h.$
  (e) Lagrange coefficient $\lambda_{i}=\prod_{j=0\cap j\neq i}^{t-1}\frac{H_{1}\left({ID}_{j}\right)}{H_{1}\left({ID}_{j}\right)-H_{1}\left({ID}_{i}\right)}$.
  (f) $\delta_{i}$ is the additive share of $\sum_{i=0}^{t-1}k_{i}^{\prime }\sum_{i=0}^{t-1}\gamma_{j},\delta_{i}=k_{i}^{\prime }\gamma_{i}+\sum_{j=0\cap j\neq i}^{t-1}\alpha_{ij}+\sum_{j=0\cap j\neq i}^{t-1}\beta_{ij}$, $k_{i}^{\prime }\gamma_{j}=\alpha_{ij}+\beta_{ji}$
  (g) ${KGC}_{i}$ broadcasts $\delta_{i}$ and computes ${ds}_{i}={(\sum_{i=0}^{t-1}\delta_{i})}^{-1}\gamma_{i}hP_{1}$
  (h) ${KGC}_{i}$ sends ${ds}_{i}$ to Combine Center.
3. Combine private key
  (a) Combine Center computes user private key $ds=P_{1}-\sum_{i=0}^{t-1}{ds}_{i}=P_{1}-{\left(\sum_{i=0}^{t-1}\delta_{i}\right)}^{-1}\sum_{i=0}^{t-1}\gamma_{i}hP_{1}=P_{1}-k^{-1}hP_{1}=P_{1}-{\left(msk+h\right)}^{-1}hP_{1}$.

### 3.5. Paillier Homomorphic Share Transformation Protocol Optimization

In the existing homomorphic share transformation protocol, two entities, Alice and Bob, possess multiplicative shares that collectively satisfy the equation x=ab mod n. This protocol can help Alice and Bob realize that they hold additive share secret α and β, respectively, satisfying α+β=x.

The number of secret random numbers that any two participants require to perform a share transformation in our scheme is two. Assuming that the secret random numbers of Alice and Bob are $a_{1},a_{2}\;and\;b_{1},b_{2}$, respectively, the final state that must be achieved is $\alpha +\beta =a_{1}b_{2}+a_{2}b_{1}$. In our scheme, this refers to the principle that “$\alpha_{ij}+\beta_{ji}=k_{i}^{\prime }\gamma_{j}+k_{j}^{\prime }\gamma_{i}$.” This signifies a specific operational relationship or mathematical model integral to the proposed scheme, which can be achieved if the previous homomorphic share transformation protocol is directly invoked twice, but it is not efficient. The reason is that two homomorphic encryption operations, one homomorphic decryption operation, and two rounds of communication are required to call this protocol once. If n participants must perform two–two secret random number share transformation, then for a total of 2n(n − 1) homomorphic encryption operations, n(n − 1) decryption operations, and 2n(n − 1) rounds of communication, the optimization scheme is specified using the following steps.

- Alice computes $A_{1}=a_{1}+a_{2},A_{2}=a_{1}-a_{2},c_{1}={Enc}_{pk}\left(A_{2}\right)$, then sends $c_{1},A_{2}$ to Bob ($where\;A_{2}$ denote the sum of two random numbers and does not reveal the specific value of the random number).
- Bob computes $B_{1}=b_{1}+b_{2},B_{2}=b_{1}-b_{2},c_{2}={c_{1}}^{B_{2}}*{Enc}_{pk}\left(r\right)={Enc}_{pk}\left(A_{2}B_{2}+r\right),r\in_{R}Z_{n}$, $D=A_{1}B_{1}$. Bob computes the additive share secret as $\beta =-\frac{r+D}{2}$ and sends $c_{2}$ to Alice.
- Alice decrypts $c_{2}$ and computes Alice’s secret share $\alpha =-\frac{Dec\left(c_{2}\right)}{2}=-\frac{A_{2}B_{2}+r}{2}$.

**Correctness Proof:**  (5) $$\begin{array}{c} \;\;\;\alpha +\beta \\ =-\frac{A_{2}B_{2}+r}{2}+\frac{r+D}{2}\\ =-\frac{A_{2}B_{2}+r}{2}+\frac{A_{1}B_{1}+r}{2}\\ =\frac{A_{1}B_{1}-A_{2}B_{2}}{2}\\ =\frac{\left(a_{1}+a_{2}\right)\left(b_{1}+b_{2}\right)-\left(a_{1}-a_{2}\right)\left(b_{1}-b_{2}\right)}{2}\\ =a_{1}b_{2}+a_{2}b_{1} \end{array}$$

□

Implementing the above protocol involves only $n\left(n-1\right)$ homomorphic encryption operations, $\frac{n\left(n-1\right)}{2}$ decryption operations, and $n\left(n-1\right)$ rounds of communication. By incorporating the finite-domain addition and division (which can also be achieved by the inverse of multiplication) computations at a lower cost, the cost is reduced significantly.

### 3.6. Scheme Correctness

The correctness of our scheme can be proved as follows:

(6) $$\begin{array}{c} \;\;ds=P_{1}-\sum_{i=0}^{t-1}{ds}_{i}\\ =P_{1}-{\left(\sum_{i=0}^{t-1}\delta_{i}\right)}^{-1}\sum_{i=0}^{t-1}\gamma_{i}hP_{1}\\ =P_{1}-{\left(\sum_{i=0}^{t-1}{k^{\prime }}_{i}\gamma_{i}+\sum_{i\neq j}\alpha_{ij}+\sum_{i\neq j}\beta_{ij}\right)}^{-1}\sum_{i=0}^{t-1}\gamma_{i}hP_{1}\\ =P_{1}-{\left(\sum_{i=0}^{t-1}{k^{\prime }}_{i}\sum_{j=0}^{t-1}\gamma_{j}\right)}^{-1}\sum_{i=0}^{t-1}\gamma_{i}hP_{1}\\ =P_{1}-{\left(\sum_{i=0}^{t-1}{k^{\prime }}_{i}\right)}^{-1}hP_{1}\\ =P_{1}-{\left(\sum_{i=0}^{t-1}\lambda_{i}k_{i}+\frac{h}{t}\right)}^{-1}hP_{1}\\ =P_{1}-k^{-1}hP_{1}\\ =P_{1}-{\left(msk+h\right)}^{-1}hP_{1}\\ =\frac{msk}{msk+h}P_{1} \end{array}$$

### 3.7. Security Analysis

In the absence of the master private key (msk), the challenger can forge a legitimate user signature private key (ds) for the user ID in polynomial time, which is negligible in probability.

**Theorem 1.**  *Supposing a random oracle, if the τ-BCAA1 assumption is hard, then our scheme is secure in the EUF-CIA model*.

**Proof:**  If there exists an adversary A that wins the EUF-CIA model under our distributed SM9 signature private key generation algorithm with advantage ϵ and A performs at most $q_{E}>0$ distributed extract query and $q_{H}>0\;H_{1}$ query, then we can construct a simulator S to solve the τ-BCAA1 problem with advantage $\frac{\epsilon }{eq_{H}}$. S simulates honest KGCs and Combine Center based on τ-BCAA1 assumption instance. To make this security certificate more representative, we assume that the semi-honest KGCs number fixed by an adversary is $\left|A\right|=t-1$ and S simulates only one KGC.

1. Setup
  S and ***A*** co-run the distributed setup algorithm
  (a) S and A obtain the generators $P_{1},P_{2}$ of the cyclic group $G_{1},G_{2}$ of the system parameters and Paillier key pair $\left({pk}_{i},{sk}_{I}\right)$, respectively.
  (b) A adaptively randomly chooses $s_{i}\in_{R}Z_{N}^{*}$ as ${msk}_{I}$, i∈A, sends $f_{i}\left(x_{j}\right)$ to corresponding KGCs and broadcast commits $C_{I,j}$, which $C_{I,0}={mpk}_{i}$.
  (c) S simulates KGC, computes ${mpk}_{S}=\left[\alpha \right]P_{2}-\sum C_{i0}=C_{S,0}$ as master public key share, then randomly chooses $r_{j}\in_{R}Z_{N}^{*}\left(j\in \left[1,t-1\right]\right)$ and sends $r_{j}$ to ${KGC}_{i}\left(i\in A\right)$.
  (d) S simulates coefficients commits,
    x11      x12⋯x1t−1x21      x22 x2t−1⋮⋱⋮xt−11      xt−12⋯xt−1t−1CS,1Cs,2⋮Cs,t−1=r1∗P2−mpkSr2∗P2−mpkS⋮rt−1∗P2−mpkS
  The matrix in the above equation is a Vandermonde matrix. Obviously the determinant is not equal to 0, and the elliptic curve points are capable of addition, subtraction and number multiplication operations, so that $C_{S,i}\left(i\in \left[1,t-1\right]\right)$ can be found by the above equation, S broadcasts $C_{S,i}$ to open Feldman Commit.
  (e) adversary can compute $mpk=\sum C_{i,0}=\left[\alpha \right]P_{2}$.
2. $H_{1}$ query
  (a) S maintains a list ${H_{1}}^{list}$ of tuples (${ID}_{I},y_{I},D_{i})$ as explained below. When A makes $H_{1}$ query on ${ID}_{I}$ that are adaptively chosen by itself, S responds as follows:
  (b) If ${ID}_{I}$ is on the list ${H_{1}}^{list}$ of tuples (${ID}_{I},y_{I},D_{i})$, S responds with $H_{1}\left({ID}_{i}\right)=y_{i}$. Otherwise, if the query is on the I-th distinct ID, then S stores (${ID}_{I},h_{0},⊥)$.
  (c) Otherwise, S selects a random integer $h_{i}\left(0<i\leq \tau \right)$ from the τ-BCAA1 instance and the instance has been not been chosen by ***S***, then ***S*** stores tuple (${ID}_{I},h_{I},[\frac{\alpha }{h_{i}+\alpha }]P_{1})$ and responds with $H_{1}\left({ID}_{i}\right)=h_{i}$.
3. Distributed private key extract query
  (a) ***A*** runs Distributed Private Key Extract algorithms and Combine Private Key algorithms with S,S selects a random integer $k_{S}\in_{R}Z_{N}^{*}$ as t threshold-private key share.
  (b) ***A*** makes private key extract queries on identities ${ID}_{I}$ that are adaptively chosen, ***S*** responds as follows:
  (c) If i=I, then ***S*** aborts the game. Otherwise, if i≠I, then there is a tuple $\left({ID}_{I},h_{I},[\frac{\alpha }{h_{i}+\alpha }]P_{1}\right)$, S responds with ${ds}_{S}$ where ${ds}_{S}=P_{1}-\sum {ds}_{i\in A}-\left[\frac{\alpha }{h_{i}+\alpha }\right]P_{1}$.
4. Forgery

A outputs forged distributed private key shares ${ds}_{i\in \left[0,t-1\right]}$ on ${ID}_{i}$. **If** i≠I, then ***S*** aborts the game. Otherwise, if i=I, ***S*** computes $\;ds=P_{1}-\sum {ds}_{i\in \left[0,t-1\right]}$ and outputs $e\left(ds,P_{2}\right)$ as a solution of τ-BCAA1 instance. □

**Claim 1.**  *If **S** does not abort during the simulation, then algorithm A’s view is identical to its view in real attack*.

**Proof:**  ***S***’s responses to $H_{1}\;$ queries are random selections of the τ-BCAA1 instance. ***S***’s responses to distributed private key queries are valid private key shares of ID which are adaptively chosen by ***A***. Those responses are uniformly independently distributed as in a real attack. □

**Claim 2.**  *If the advantage* ϵ *of winning the game is the probability of returning valid forged private key shares *${ds}_{i\in \left[0,t-1\right]}$*, then **S** is able to solve τ-BCAA1 problem with advantage *$\frac{\epsilon }{eq_{H}}$.

**Proof:**  The success of ***S*** in resolving τ-BCAA1 problem is determined by the following three events:

Event1: ***S*** does not abort during the Distributed Private Key Extract query.

Event2: ***A*** forges valid private key shares ${ds}_{i\in \left[0,t-1\right]}$ on ID.

Event3: Event2 occurs and index of the tuple ${(ID}_{i},y_{i},D_{i})$ corresponding to the ID is i=I.

$\mathrm{Pr}\left[Event1\right]$ = ${\left(1-\frac{1}{q_{E}}\right)}^{q_{E}}$,

$\mathrm{Pr}\left[Event2|Event1\right]$ = ϵ

$\mathrm{Pr}\left[Event3|Event1\wedge Event2\right]$ = $\frac{1}{q_{H}}$

Thus, the advantage of ***S*** in solving τ-BCAA1 problem is as follows:

$$\begin{array}{cc} {Adv}_{S}^{\tau -BCAA1} & =Pr\left[Event1\wedge Event3\right]\\ & =Pr\left[Event1\right]Pr\left[Event2|Event1\right]Pr\left[Event3|Event1\wedge Event2\right]\\ & ={\left(1-\frac{1}{q_{E}}\right)}^{q_{E}}\frac{1}{q_{H}}\epsilon \approx \frac{\epsilon }{eq_{H}} \end{array}$$

□

## 4. Performance Evaluation

### 4.1. Theoretical Analysis

Our scheme enhances the original homomorphic share transformation protocol, with a focus on achieving substantial improvements in computational and communication efficiency. The following table shows the main system cost after invoking various share transformation protocols when t KGCs perform DKG.

The original scheme in Table 2 is based on the study by Tu et al. [30]. Here, we highlight some critical details of the proposed scheme. Following the SM9 digital signature algorithm specifications, we selected a 256-bit prime number as the cyclic group’s order. This guarantees multiplicative inverses for all coefficients in the cyclic group, thereby facilitating the integration of Shamir’s secret sharing into our scheme. However, in the Paillier share conversion protocol, the computation involves number m∈Zn, where n is the product of two primes p and q, which do not directly correspond to the cyclic group’s order N in the SM9 algorithm. To prevent computational errors due to cyclic group order discrepancies, p and q in the Paillier protocol are selected as 257-bit primes, exceeding twice N’s size. The choice is pivotal, as both Paillier protocols necessitate homomorphic multiplication and addition, i.e., ${Enc\left(a\right)}^{b}*Enc\left(c\right)=Enc\left(ab+c\right)$ for $a,b,c\in_{R}Z_{N}$. Consequently, n is a 514-bit, and the Paillier ciphertext is a 1028-bit random number. Table 2 shows that in the original scheme, each of t KGCs performs t Paillier encryptions, stores ciphertexts (1028 bits) and plaintexts (256 bits), receives *t* − 1 ciphertexts, and conducts decryption. Implementing the Paillier protocol yields *t* − 1 intermediate variable $r\in_{R}Z_{N}$ (256 bits), necessitating a storage total of $\left(2t^{2}-2t\right)1028\;bits+\left(2t^{2}-t\right)256\;bits$ in the original protocol. Beyond computational overhead, each KGC executes *t* − 1 homomorphic additions and multiplications, incurring a computational cost of $t^{2}T_{Enc}+\left(t^{2}-t\right)T_{Dec}+\left(t^{2}-t\right)T_{HomAdd}+\left(t^{2}-t\right)T_{HomMul}$. In our improvement scheme, *t* KGCs undertake (*t* + 1)/2 Paillier encryptions and store ciphertexts (1028 bits) and plaintexts, and the latter is computed from a 256-bit constant and a random number. Additionally, they obtain (*t* − 1)/2 ciphertexts and a 256-bit random number, with the Paillier protocol generating (*t* − 1)/2 intermediates r for storage, culminating in a total of $\frac{{2t}^{2}+t}{2}1028\;bits+2t^{2}256\;bits$ storage in the enhanced scheme. Exceeding the aforementioned computational cost, each KGC conducts (*t* − 1)/2 homomorphic additions and multiplications, accruing a total cost of $\frac{t^{2}+t}{2}T_{Enc}+\frac{t^{2}-t}{2}T_{Dec}+\frac{t^{2}-t}{2}T_{HomAdd}+\frac{t^{2}–t}{2}T_{HomMul}$, where $T_{Enc}$ denotes the encryption operation time, $T_{Dec}$ denotes the decryption operation time, $T_{HomAdd}$ denotes the homomorphic addition operation time, and $T_{HomMul}$ denotes the homomorphic multiplication operation time. From Table 2, it can be observed that our scheme has significantly improved storage space, computational time overhead, and communication rounds compared to the original scheme.

### 4.2. Experimental Analysis

**Figure 3:**
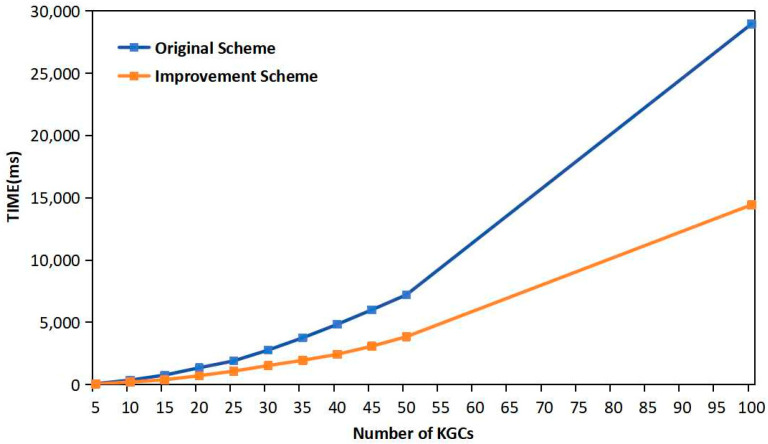
Comparison of scheme time consumption.

Experimental validation for this study was conducted using a computer with a 3.0 GHz 6-core 64-bit AMD Ryzen 5 4600H processor and 16 GB of RAM, operating on a Windows 10 platform. The experimental design employed the Java Pairing-Based Cryptography (JPBC) library, adhering to the stringent SM9 standard recommendations for algorithm parameters. This adherence is pivotal, as it ensures that our findings hold practical significance in the field of the identification and verification of cryptographic algorithms. As shown in Figure 3 and Table 3, by simulating the operational environment, the experiments were designed to emulate the implementation of multiple KGCs as part of a DKG system. The empirical evidence gleaned from these simulations offers a robust corroboration of the theoretical performance analysis previously detailed in this study. The results unambiguously affirm that our improved scheme aligns with theoretical predictions and provides a considerable enhancement in performance when compared to the original scheme.

The graphical data presented below detail a side-by-side comparison of the “Original Scheme” and “Improved Scheme” with an increasing number of KGCs. The original scheme is illustrated by an orange dashed trajectory, revealing a pronounced upward trend in computational time, which suggests scalability concerns. Conversely, the “Improved Scheme”, depicted by a steady blue line, indicates a tempered ascent in time with additional KGCs, illustrating a more scalable solution. The tabular data echo the graphical insights, clearly quantifying the differences in milliseconds (ms) across a spectrum of 5–100 KGCs, which reinforces the performance benefits of the improved scheme.

Notably, the combination of both graphical and tabular data encapsulated within these images provides a transparent and quantifiable assessment of the efficacy of the cryptographic schemes in question. The enhancement offered by the refined scheme is substantial, setting a new benchmark for system efficiency and scalability in cryptographic practices.

## 5. Conclusions

To address the challenges posed by the prohibitive cost of DKG within the SM9 framework, this study introduces an optimized approach called the SM9-based Lightweight Distributed Key Generation Scheme, which operates effectively in the absence of trusted centers. Through rigorous analysis, we successfully demonstrated the robust security credentials of the scheme within the EUF-CIA model, substantiating its resilience by demonstrating a reduction in the τ-BCAA1 problem under the random oracle model. Empirical evaluations further underscore the substantial enhancements in efficiency offered by our scheme, demonstrating a significant step forward in the practical deployment of distributed cryptographic solutions. However, a notable limitation of our proposed scheme is that it is constructed under the assumption of a semi-honest adversary. Future research will explore the utilization of zero-knowledge proofs and related technologies to develop a secure scheme under the assumption of malicious adversaries. This progression will enhance the applicability of our approach in more demanding scenarios where adversarial behavior may compromise security.

## Figures and Tables

**Table 1:** Distributed key generation protocol based on SM9.

Scheme	Number of Parties	(t,n) Threshold	Relationship Between t and n	System Cost
Ma [28]	Multi-party	√	n ≥ 2t−1	Moderate
Zhang et al. [25]	Multi-party	√	n ≥ 2t−1	Moderate
Tu et al. [29]	Multi-party	√	n ≥ t	High
Yu et al. [30]	Two-party	×	n = t = 2	Low

**Table 2:** Comparison of system cost.

Scheme	Storage Space (bits)	Computational Time	Communication Rounds
Original scheme	$\left(2t^{2}-2t\right)1028\;bits\;+\left(2t^{2}-t\right)256\;bits$	$t^{2}T_{Enc}+\left(t^{2}-t\right)T_{Dec}\;+\left(t^{2}-t\right)T_{HomAdd}\;+\left(t^{2}-t\right)T_{HomMul}$	$t^{2}-t$
Improvement scheme	$\frac{{2t}^{2}+t}{2}1028\;bits\;+2t^{2}256\;bits$	$\frac{t^{2}+t}{2}T_{Enc}+\frac{t^{2}-t}{2}T_{Dec}+\;\frac{t^{2}-t}{2}T_{HomAdd}\;+\frac{t^{2}-t}{2}T_{HomMul}$	$\frac{t^{2}-t}{2}$

**Table 3:** Comparison of scheme time consumption.

Scheme Number of KGCs	Original Scheme	Improvement Scheme
5	82 ms	44 ms
10	365 ms	207 ms
15	768 ms	390 ms
20	1358 ms	721 ms
25	1909 ms	1087 ms
30	2781 ms	1536 ms
35	3767 ms	1957 ms
40	4842 ms	2437 ms
45	6009 ms	3086 ms
50	7212 ms	3850 ms
100	28,970 ms	14,441 ms

## Data Availability

The data are contained within the article.
